# Engineering glyphosate‐resistant watermelon by prime editing

**DOI:** 10.1111/jipb.70344

**Published:** 2026-07-06

**Authors:** Shouwei Tian, Yu Lu, Jingchao Chen, Guoyi Gong, Haiying Zhang, Jie Zhang, Changlong Wen, Yong Xu

**Affiliations:** ^1^ State Key Laboratory of Vegetable Biobreeding, National Engineering Research Center for Vegetables, Beijing Key Laboratory of Crop Molecular Design and Intelligent Breeding, Beijing Key Laboratory of Vegetable Germplasms Improvement, Beijing Vegetable Research Center Beijing Academy of Agriculture and Forestry Science Beijing 100097 China; ^2^ State Key Laboratory of Plant Environmental Resilience, College of Biological Sciences China Agricultural University Beijing 100193 China; ^3^ State Key Laboratory for Biology of Plant Diseases and Insect Pests, Institute of Plant Protection Chinese Academy of Agricultural Sciences Beijing 100193 China

## Abstract

Editing the watermelon *EPSPS* gene using a prime editing platform with visible markers created a non‐transgenic glyphosate‐resistant line. These robust heterozygous mutants tolerate field‐level herbicide doses without growth penalties, serving as ideal parental lines for crop breeding.

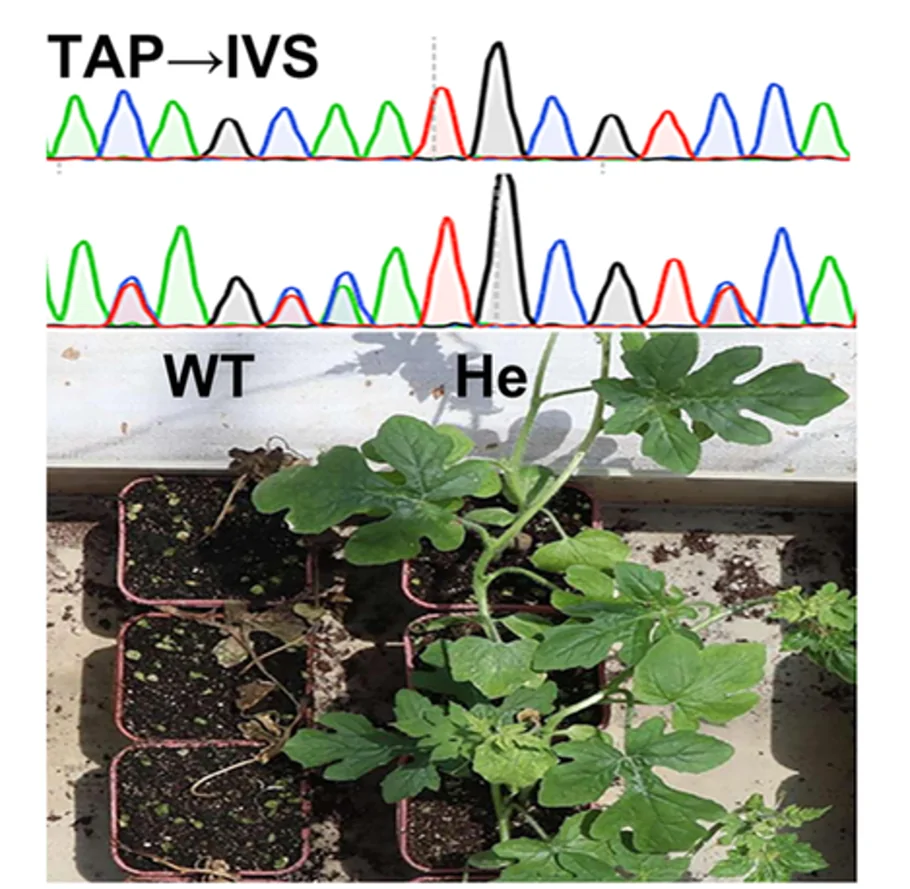

Watermelon (*Citrullus lanatus*) is a globally cultivated crop whose wide‐row planting pattern often leads to severe weed infestation, significantly reducing yield. While previous base editing efforts developed germplasm resistant to acetolactate synthase (ALS)‐inhibiting herbicides (Tian et al., 2018), their prolonged sole use risks weed resistance and soil residue accumulation. Thus, creating novel glyphosate‐resistant varieties is crucial. Glyphosate targets 5‐enolpyruvylshikimate‐3‐phosphate synthase (EPSPS) and provides broad‐spectrum control with favorable environmental compatibility, supporting sustainable weed management.

The *EPSPS* TAP‐IVS triple mutation (Thr102→Ile, Ala103→Pro, Pro106→Ser) confers robust glyphosate resistance while maintaining native catalytic activity (Perotti et al., 2019). Introducing this mutation requires precise simultaneous multi‐base substitutions, making prime editing (PE) an ideal tool (Anzalone et al., 2019). PE has been successfully applied in monocots (Lin et al., 2020), including the creation of glyphosate‐resistant germplasm (Qiao et al., 2023), though comprehensive evaluation of such resistance in new germplasm remains limited. Furthermore, while PE has been reported in model dicots like tomato and Arabidopsis (Vu et al., 2024), its efficiency in most other dicot crops remains impractically low, and crucially, it has never been established in watermelon or other cucurbits. In this study, by developing a rapid visual assay to optimize PE components, we established the first functional prime editing platform in watermelon. We precisely introduced the *TAP‐IVS* triple mutation into *EPSPS*, creating non‐transgenic glyphosate‐resistant germplasm and providing a versatile precision breeding toolkit for cucurbits.

Developing a rapid evaluation method is crucial for establishing an efficient PE system. However, the protoplast‐based approach in watermelon is technically challenging due to the difficulty in obtaining explants suitable for protoplast isolation and the lack of an efficient transformation protocol. To overcome this, we constructed a visual, high‐throughput *Agrobacterium rhizogenes*‐mediated hairy root transformation system. We constructed two visual editing vectors, pBSE406 (*tdTomato*) and pBSE407 (*DsRed2*), incorporating respective fluorescent markers (Figure 1A). Using germinating seeds, positive transgenic roots were visually identified via red fluorescence within three weeks (Figures 1A, S1), with successful edits confirmed by Sanger sequencing (Figure S2). This simple, intuitive, and highly stable method provides a reliable alternative to protoplast assays for optimizing PE components.

**Figure 1:**
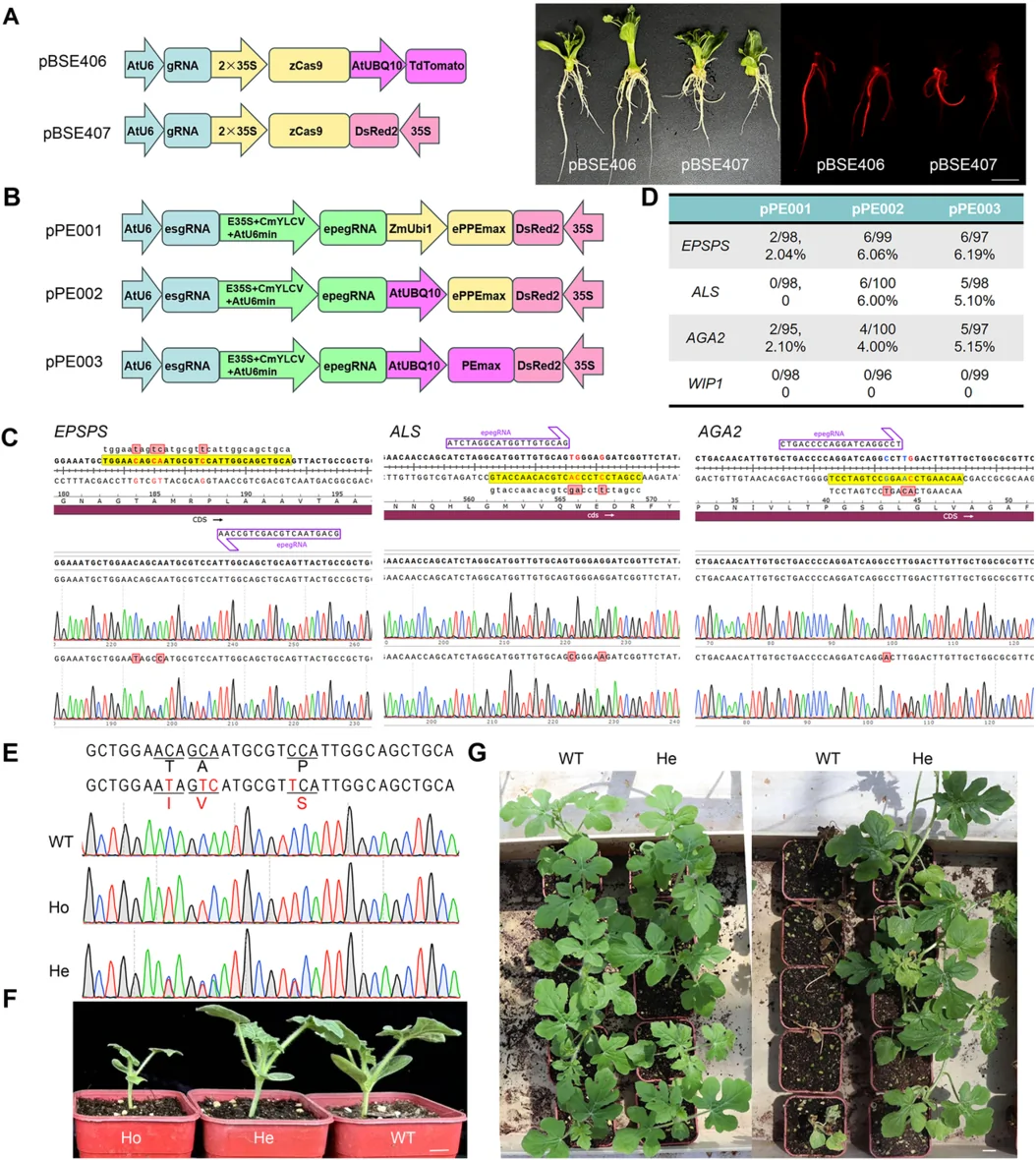
Engineering glyphosate‐resistant watermelon by prime editing **(A)** Two visual transient detection systems for highly efficient *Agrobacterium rhizogenes*‐mediated hairy root transformation for gene editing analysis in watermelon. The left panel depicts the schematic structures of the two gene‐editing vectors encoding red fluorescent protein. The right panel shows the identification of positive fluorescent hairy roots following *A. rhizogenes* infection. **(B)** Schematic diagram of the prime editing (PE) vector construct. **(C)** Sanger sequencing chromatograms confirming successful prime editing. The target base for substitution is indicated by a red letter. The top row shows the chromatogram of the wild type, and the bottom row shows the chromatogram of successfully edited plants. **(D)** Editing frequency of three PE editors in a transient hairy root system. Editing frequency is presented as the percentage of successfully edited roots (by Sanger sequencing) among all red fluorescent protein‐positive roots. **(E)** Sequencing chromatograms of the edited *EPSPS* gene (TAP‐IVS site) in T1 plants. The targeted base substitutions are highlighted in red. The corresponding amino acid changes are indicated below the respective nucleotide sequences. WT: wild type; Ho: homozygous mutant; He: heterozygous mutant. **(F)** Growth comparison of seedlings from edited *EPSPS* (TAP‐IVS) plants and wild type. **(G)** Glyphosate resistance assay of wild‐type and *EPSPS* (TAP‐IVS) heterozygous plants. Plants were treated with glyphosate at the recommended working concentration (900 g ai ha^−1^). The left panel shows plants before spraying, and the right panel shows the same plants one week after treatment. Scale bars: 2 cm.

Based on the pBSE407 backbone, which features a more compact fluorescent expression cassette than pBSE406, we assembled the watermelon PE system (Figure 1B). The prime editing guide RNA (pegRNA) utilized the enhanced epegRNA structure (Nelson et al., 2022) driven by a highly specific U6 composite promoter (Jiang et al., 2020). For the nCas9‐reverse transcriptase (nCas9‐RT) fusion, we selected two variants with demonstrated high editing efficiency in plant editing performance: ePPEmax (Ni et al., 2023) and PEmax (Qiao et al., 2023). These were driven by the strong *AtUBQ10* promoter to create vectors pPE002 and pPE003, respectively. A control vector, pPE001 (ePPEmax driven by *ZmUBi1* promoter), was also constructed to evaluate promoter suitability. All vectors included fluorescent markers and selection elements to ensure stable screening (Figure 1B).

To introduce the *TAP‐IVS* triple mutation into the *EPSPS* gene, pegRNA and nicking gRNA sequences were designed following Xu et al. (2022) and incorporated into the pPE001, pPE002, and pPE003 vectors. For systematic comparison, three endogenous genes were also targeted (Table S2). Following hairy root induction, positive roots were screened via fluorescence after two weeks. Sanger sequencing confirmed successful editing via overlapping peaks at the target sites (Figure 1C). Based on the transient assay, pPE001 showed low editing frequency (defined as the proportion of transformants carrying the desired edit) of 0–2.10%, successfully editing only two genes. In contrast, pPE002 edited three genes with up to 6.06% editing frequency, and pPE003 reached 6.19%. While pPE003 and pPE002 showed no significant difference, both significantly outperformed pPE001 (Figure 1D). Notably, one target site resisted editing across all constructs, likely due to sequence‐specific barriers such as pegRNA instability or inaccessible chromatin.

Using the optimized pPE002‐*EPSPS* and pPE003‐*EPSPS* vectors, stable *Agrobacterium tumefaciens*‐mediated transformation yielded T0 plants. pPE002 produced 47 positive seedlings with four successfully edited (8.51% editing frequency); pPE003 produced 57 positive seedlings with four edited (7.02% editing frequency). No homozygous mutants were obtained in T0 (Figure S3). To precisely quantify the allelic mutation rates, next‐generation sequencing (NGS) was performed on all T0 plants, revealing an average desired PE efficiency of 4.10% for pPE002 and 4.19% for pPE003 (Table S3). Although the editing frequency we observed is moderate compared to the maximum rates in model dicots (e.g., 38% in Arabidopsis, 26% in tomato) (Vu et al., 2024), it aligns with the common experience of significant locus‐dependent variation, where many targets in those studies also showed low or no editing. Thus, our establishment of the first functional prime editing platform in watermelon—and indeed, in any cucurbit crop—marks a significant technical advance. Success in this recalcitrant species provides a foundational system for future optimization and precise breeding within this agronomically important family.

Homozygous *TAP*‐*IVS* triple mutants isolated in the T1 generation (Figure 1E) exhibited severe phenotypic alterations, including dwarfism and complete male sterility. Furthermore, the T2 progeny of non‐transgenic heterozygous T1 plants showed highly skewed segregation (4.8% homozygous, 59.0% heterozygous, 36.2% wild‐type) (Table S4), confirming the partial lethality of the homozygous mutation. In contrast, heterozygous mutants showed no significant differences from wild‐type in growth vigor, plant height, and reproductive development (Figures 1F, S4), indicating *EPSPS* gene dosage effects. Given their normal growth and stable mutation inheritance, heterozygous mutants offer significant practical breeding potential. Because commercial watermelons are F1 hybrids, these lines serve as ideal parents for developing glyphosate‐resistant varieties. In practice, these heterozygous individuals can be efficiently tracked and isolated using Kompetitive Allele Specific PCR (KASP) markers. Following glyphosate application at the recommended field dose (1×, 900 g ai ha^−1^), all wild‐type plants died within seven days, whereas heterozygous mutants grew robustly (Figure 1G). Crucially, key agronomic traits remained unaffected following the herbicide treatment (Table S5). In dose‐gradient experiments, heterozygous mutants survived up to an 8× dose, while wild‐type plants perished at half the recommended dose (Figure S5).

In summary, establishing the first prime editing platform in watermelon represents a crucial step for precision breeding in cucurbit crops. We complemented this system with a robust visual assay for the rapid evaluation and optimization of editing components. By precisely engineering the *EPSPS* gene, we created novel glyphosate‐resistant germplasm. The resulting heterozygous mutants, which exhibit robust herbicide resistance without agronomic penalties, provide immediately valuable parental material. While future optimization of PE proteins and promoters will further augment editing frequencies, this current achievement provides a transformative toolkit for watermelon improvement and establishes a cornerstone for precise genome engineering across the Cucurbitaceae family.

## CONFLICTS OF INTEREST

The authors declare no conflicts of interest.

## AUTHOR CONTRIBUTIONS

S.T. and Y.X. designed the research. S.T., Y.L., and J.C. performed the experiments. Other authors analyzed and interpreted the data. S.T. and Y.X. drafted the manuscript. All authors have read and approved the final manuscript.

## Supporting information

Additional Supporting Information may be found online in the supporting information tab for this article: http://onlinelibrary.wiley.com/doi/10.1111/jipb.70344/suppinfo

**Figure S1.** ***Agrobacterium rhizogenes*** **‐mediated rooting protocol in watermelon.** Explants were treated with ultrasound, infected with *A. rhizogenes*, and co‐cultivated. After selection on glufosinate ammonium‐containing medium, editing efficiency was evaluated by red fluorescence in hairy roots.
**Figure S2. Schematic diagram of editing efficiency evaluation based on Sanger sequencing of fluorescent roots.** The PAM sequence is shown in blue, the gRNA target site is underlined in purple, and overlapping chromatogram peaks indicate the presence of edited sequences.
**Figure S3. Sanger sequencing chromatograms of the eight edited T0 watermelon plants.** The chromatograms display the targeted EPSPS genomic region in the eight independent T0 plants generated using the pPE002 (pPE002‐T0‐1 to 4) and pPE003 (pPE003‐T0‐1 to 4) prime editing vectors. The wild‐type reference nucleotide sequence and its corresponding amino acid sequence are shown at the top.
**Figure S4. Growth comparison between EPSPS heterozygous mutants and the wild type.** Panel A depicts the growth status of EPSPS heterozygous mutants versus the wild‐type. Panel B presents a quantitative analysis of fruit weight and seed number per fruit for both groups, with a t‐test analysis showing no significant differences.
**Figure S5. Resistance test to glyphosate in EPSPS heterozygous mutants vs. wild type.** The front row shows wild‐type control plants, and the back row shows *epsps* heterozygous plants. Each plant was treated with the concentration of glyphosate indicated on its pot.
Table S1. DNA sequences of related vectors. Table S2. Prime editing targets sequences used in this study.
Table S3. Next‐generation sequencing (NGS) analysis of desired prime editing (PE) efficiency in T0 transgenic plants.
Table S4 Genotype segregation of the T2 progeny derived from non‐transgenic heterozygous T1 plants.
Table S5 Effects of prime editing and glyphosate treatment on key agronomic traits. Table S6. Primers for amplification of prime editing targets in this study.
