# Supplementary material for: Engineering glyphosate‐resistant watermelon by prime editing
Source: J Integr Plant Biol. 2026 Jul 6;68(9):3298–300. doi: 10.1111/jipb.70344 (PMC13545817; doi:10.1111/jipb.70344)
Supplement: Supplementary file 1 [file JIPB-68-3298-s001.docx]

**Engineering Glyphosate-Resistant Watermelon by Prime Editing**
Shouwei Tian^1^, Yu Lu^2^, Jingchao Chen^3^, Guoyi Gong^1^, Haiying Zhang^1^, Jie Zhang^1^, Changlong Wen^1^, Yong Xu^1^

**Supplementary methods**

**Plant materials and growth conditions**

The watermelon inbred line 'ZZJM' used in this study was developed by the Watermelon Breeding Group at the Beijing Academy of Agriculture and Forestry Sciences. Tissue-cultured seedlings were grown on Murashige and Skoog (MS) medium, which was customized by Coolaber (Beijing, China). The medium contained 30% (w/v) sucrose and was solidified with 0.5% (w/v) agar, with its pH adjusted to 5.8 . All plants were maintained under controlled greenhouse conditions with a 16 h/8 h light/dark cycle at a constant temperature of 25℃-28℃.

**Vector construction**

The PBSE406 and PBSE407 vectors were derived from the PBSE401 backbone (Xing et al. 2014). Specifically, the PBSE401 plasmid was linearized by single-enzyme digestion with EcoRI, and the resulting fragment was used for recombinase-mediated assembly with the TdTomato and DsRed2 expression cassettes to create PBSE406 and PBSE407, respectively.
 The PE001-PE003 vectors were all constructed using PBSE407 as the backbone. These vectors retained the original antibiotic resistance gene and the fluorescent protein expression cassette. The gRNA and Cas9 coding sequences in the backbone were replaced with the corresponding key components for prime editing via a homologous recombination method, as schematically illustrated in Figure 1C. The DNA fragments encoding these key components for the PE vectors were synthesized by General Biol (China). The sequences of all related vector elements mentioned above are provided in Table S1. Information regarding the target sites involved in this study can be found in Table S2.

**A.hizogenes K599-Mediated Hairy Root Transformation in Watermelon**

The PE vectors were introduced into Agrobacterium rhizogenes strain K599 using the freeze-thaw method. Germinated watermelon seeds were prepared by excising the emerging radicles. These explants were placed in a conical flask containing 20 ml of liquid MS medium. The flask was sealed and subjected to ultrasonic treatment for 15 seconds to increase wounding on the explants. The corresponding A. rhizogenes infection suspension was added, and the flask was shaken for 30 minutes before the bacterial suspension was discarded. After one rinse with sterile water, the seeds were sown onto solid MS medium in transparent glass jars and co-cultivated in the dark for 2-3 days. Subsequently, the seeds were transferred to solid MS medium supplemented with timentin (250 mg/L) and glufosinate ammonium (3 mg/L) and grown under standard light conditions. Two weeks later, editing efficiency was assessed by detecting red fluorescence in the emerging hairy roots under ultraviolet light.

**Agrobacterium-Mediated Transformation in Watermelon**

The Agrobacterium tumefaciens-mediated transformation of watermelon (variety 'ZZJM') was performed using the GV3101 strain, following a previously described method (Tian et al. 2017). Cotyledonary nodes excised from watermelon seedlings were co-cultivated with Agrobacterium carrying the respective binary vectors, which were introduced into the bacterium via the freeze-thaw method. Following co-cultivation, the explants were transferred to a selection medium containing 3 mg L⁻¹ glufosinate ammonium and cultured for four weeks to isolate resistant tissues. Vigorously growing, herbicide-resistant tissues were subsequently transferred to a regeneration medium to promote the development of transgenic plantlets. Successful transformation was visually confirmed by red fluorescence in the regenerated plantlets, which served as a basis for subsequent molecular analyses.

**DNA Extraction and analysis of prime editing in Watermelon Plants**

Genomic DNA was extracted from transgenic positive roots and plants using the CTAB method. The target sequences were amplified with specific primers (see Table S6), and the resulting PCR products were sent to Tsingke Biological Technology for Sanger sequencing. The sequencing chromatograms of each target site were analyzed using SnapGene software. The depth of NGS sequencing is about 10 k reads for each PCR amplicon in T0 plants. We analyzed NGS sequencing results by Hi-TOM assay with 5% threshold.

**Assessment of Glyphosate Resistance in watermelon**

The seeds germinated in plastic pots (8×8 cm) filled with soil matrix and organic fertilizer (volume ratio of 3:1). All the plants were kept in a greenhouse (Beijing, China) with a temperature ranging from 25 to 35℃. A series doses of glyphosate were designed based on the recommend dose which shown in Fig. S4. Glyphosate was sprayed using a mobile spray bar equipped with a flat fan nozzle (TeeJet® XR8002) compressed air cabinet sprayer (3WPSH-500D) when the plants reached the 3–5-leaf stage.

**Generative AI Usage Statement**

During the preparation of this work, the authors utilized Gemini for the purpose of enhancing the linguistic fluency and readability of their manuscript. After using this service, the authors carefully reviewed and revised the content where necessary, and take full responsibility for the final version of the publication.

**REFERENCE**

Tian S, Jiang L, Gao Q, Zhang J, Zong M, Zhang H, Ren Y, Guo S, Gong G, Liu F, Xu Y (2017) Efficient CRISPR/Cas9-based gene knockout in watermelon. **Plant Cell Rep** 36: 399-406

Xing HL, Dong L, Wang ZP, Zhang HY, Han CY, Liu B, Wang XC, Chen QJ (2014) A CRISPR/Cas9 toolkit for multiplex genome editing in plants. **BMC Plant Biol** 14: 327

**Figure S1. *Agrobacterium rhizogenes*-Mediated Rooting Protocol in Watermelon.** Explants were treated with ultrasound, infected with *A. rhizogenes*, and co-cultivated. After selection on glufosinate ammonium-containing medium, editing efficiency was evaluated by red fluorescence in hairy roots.

| **ID** | **Target Sequence** | **Fluorescent Root Count** | **Edited Root Count** | **Editing Efficiency** |
| --- | --- | --- | --- | --- |
| gRNA1 | TTCGAGGTTGGTCTTAGCA | 22 | 16 | 72.72% |
| gRNA2 | GTCGCTTGTGCTAATACGT | 22 | 13 | 59.09% |

**Figure S2. Schematic Diagram of Editing Efficiency Evaluation Based on Sanger Sequencing of Fluorescent Roots.** The PAM sequence is shown in blue, the gRNA target site is underlined in purple, and overlapping chromatogram peaks indicate the presence of edited sequences.

**Figure S3. Sanger sequencing chromatograms of the eight edited T0 watermelon plants.** The chromatograms display the targeted EPSPS genomic region in the eight independent T0 plants generated using the pPE002 (pPE002-T0-1 to 4) and pPE003 (pPE003-T0-1 to 4) prime editing vectors. The wild-type reference nucleotide sequence and its corresponding amino acid sequence are shown at the top.

**Figure S4. Growth Comparison Between EPSPS Heterozygous Mutants and the Wild Type.** Panel A depicts the growth status of EPSPS heterozygous mutants versus the wild-type. Panel B presents a quantitative analysis of fruit weight and seed number per fruit for both groups, with t-test analysis showing no significant differences.

**Figure S5. Resistance Test to Glyphosate in EPSPS Heterozygous Mutants vs. Wild Type.** The front row shows wild-type control plants, and the back row shows *epsps* heterozygous plants. Each plant was treated with the concentration of glyphosate indicated on its pot.

Table S1. DNA sequences of related vectors

>tdTomato

ATGAAGACCAACCTGTTCCTGTTCCTGATCTTCTCACTGCTGCTCAGCCTCAGCAGCGCCGAGTTCGTGTCTAAGGGTGAGGAGGTCATCAAGGAGTTCATGAGATTCAAGGTCAGGATGGAGGGTAGCATGAACGGACACGAGTTCGAGATCGAGGGTGAGGGTGAGGGTAGACCTTACGAGGGAACCCAGACCGCTAAGCTCAAGGTCACTAAGGGTGGACCACTCCCATTCGCTTGGGACATCCTCAGCCCTCAGTTCATGTACGGAAGCAAGGCTTACGTCAAGCACCCTGCTGACATCCCCGACTACAAGAAGCTCAGCTTCCCCGAGGGATTCAAGTGGGAGAGAGTCATGAACTTCGAGGACGGAGGACTCGTCACTGTCACCCAGGACAGCAGCCTCCAGGACGGAACCCTCATCTACAAGGTCAAGATGAGAGGAACCAACTTCCCACCCGACGGACCTGTCATGCAGAAGAAGACTATGGGATGGGAGGCTAGCACCGAGAGACTCTACCCCAGAGACGGAGTCCTCAAGGGTGAGATCCACCAGGCTCTCAAGCTCAAGGACGGAGGACACTACCTCGTCGAGTTCAAGACCATCTACATGGCTAAGAAGCCTGTCCAGCTCCCTGGATACTACTACGTGGACACCAAGCTCGACATCACTAGCCACAACGAGGACTACACTATCGTCGAGCAGTACGAGAGATCAGAGGGTAGACACCACCTGTTCCTCGGACACGGCACTGGATCTACTGGTAGCGGATCTAGCGGTACTGCCTCTAGCGAGGATAACAACATGGCTGTGATCAAGGAGTTCATGCGCTTCAAGGTGCGTATGGAGGGCTCTATGAATGGCCATGAGTTCGAGATCGAGGGCGAGGGCGAGGGCCGTCCATACGAGGGCACTCAGACCGCCAAGCTGAAGGTGACCAAGGGTGGCCCACTGCCATTCGCCTGGGATATCCTGTCACCACAGTTCATGTACGGCTCTAAGGCTTACGTGAAGCATCCAGCCGATATCCCAGATTACAAGAAGCTGTCATTCCCAGAGGGCTTCAAGTGGGAGCGTGTGATGAACTTCGAGGATGGTGGCCTGGTGACAGTGACCCAGGATTCATCACTGCAGGATGGCACCCTGATCTACAAGGTGAAGATGCGTGGCACCAACTTCCCACCCGATGGCCCAGTGATGCAGAAGAAGACAATGGGCTGGGAGGCTTCAACCGAGCGTCTGTACCCACGTGATGGCGTGCTGAAGGGCGAGATCCATCAGGCCCTGAAGCTGAAGGACGGTGGCCATTACCTGGTGGAGTTCAAGACAATCTACATGGCCAAGAAGCCAGTGCAGCTGCCAGGCTACTACTACGTCGATACTAAGCTGGATATCACATCACACAATGAGGATTACACCATCGTGGAGCAGTACGAGCGTTCAGAGGGCCGTCATCATCTGTTCCTGTACGGCATGGATGAGCTGTACAAGCACGACGAGCTGtga

>DsRed2

ATGGCCTCCTCCGAGAACGTCATCACCGAGTTCATGCGCTTCAAGGTGCGCATGGAGGGCACCGTGAACGGCCACGAGTTCGAGATCGAGGGCGAGGGCGAGGGCCGCCCCTACGAGGGCCACAACACCGTGAAGCTGAAGGTGACCAAGGGCGGCCCCCTGCCCTTCGCCTGGGACATCCTGTCCCCCCAGTTCCAGTACGGCTCCAAGGTGTACGTGAAGCACCCCGCCGACATCCCCGACTACAAGAAGCTGTCCTTCCCCGAGGGCTTCAAGTGGGAGCGCGTGATGAACTTCGAGGACGGCGGCGTGGCGACCGTGACCCAGGACTCCTCCCTGCAGGACGGCTGCTTCATCTACAAGGTGAAGTTCATCGGCGTGAACTTCCCCTCCGACGGCCCCGTGATGCAGAAGAAGACCATGGGCTGGGAGGCCTCCACCGAGCGCCTGTACCCCCGCGACGGCGTGCTGAAGGGCGAAACCCACAAGGCCCTGAAGCTGAAGGACGGCGGCCACTACCTGGTGGAGTTCAAGTCCATCTACATGGCCAAGAAGCCCGTGCAGCTGCCCGGCTACTACTACGTGGACGCCAAGCTGGACATCACCTCCCACAACGAGGACTACACCATCGTGGAGCAGTACGAGCGCACCGAGGGCCGCCACCACCTGTTCCTGTAG

>U6 composite promoter

CCATGGAGTCAAAGATTCAAATAGAGGACCTAACAGAACTCGCCGTAAAGACTGGCGAACAGTTCATACAGAGTCTCTTACGACTCAATGACAAGAAGAAAATCTTCGTCAACATGGTGGAGCACGACACGCTTGTCTACTCCAAAAATATCAAAGATACAGTCTCAGAAGACCAAAGGGCAATTGAGACTTTTCAACAAAGGGTAATATCCGGAAACCTCCTCGGATTCCATTGCCCAGCTATCTGTCACTTTATTGTGAAGATAGTGGAAAAGGAAGGTGGCTCCTACAAATGCCATCATTGCGATAAAGGAAAGGCCATCGTTGAAGATGCCTCTGCCGACAGTGGTCCCAAAGATGGACCCCCACCCACGAGGAGCATCGTGGAAAAAGAAGACGTTCCAACCACGTCTTCAAAGCAAGTGGATTGATGTGATTGGCAGACATACTGTCCCACAAATGAAGATGGAATCTGTAAAAGAAAACGCGTGAAATAATGCGTCTGACAAAGGTTAGGTCGGCTGCCTTTAATCAATACCAAAGTGGTCCCTACCACGATGGAAAAACTGTGCAGTCGGTTTGGCTTTTTCTGACGAACAAATAAGATTCGTGGCCGACAGGTGGGGGTCCACCATGTGAAGGCATCTTCAGACTCCAATAATGGAGCAATGACGTAAGGGCTTACGAAATAAGTAAGGGTAGTTTGGGAAATGTCCACTCACCCGTCAGTCTATAAATACTTAGCCCCTCCCTCATTGTTAAGGGAGCAAAATCTCAGAGAGATAGTCCTAGAGAGAGAAAGAGAGCAAGTAGCCTAGAAGTAGTCAAGGCGGCGAAGTATTCAGGCACGTGGCCAGGAAGAAGAAAAGCCAAGACGACGAAAACAGGTAAGAGCTAAGCATCTAGAAAGTTGAAAACAATCTTCAAAAGTCCCACATCGCTTAGATAAGAAAACGAAGCTGAGTTTATATACAGCTAGAGTCGAAGTAGTGATT

>esgRNA, tRNA-nicking targets-sgRNA
aacaaagcaccagtggtctagtggtagaatagtaccctgccacggtacagacccgggttcgattcccggctggtgcaNNNNNNNNNNNNNNNNNNNNgtttcagagctatgctggaaacagcatagcaagttgaaataaggctagtccgttatcaacttgaaaaagtggcaccgagtcggtgctttttttt

>epegRNA, tRNA-PE targets-sgRNA-RT+PBS-tevopreQ1

aacaaagcaccagtggtctagtggtagaatagtaccctgccacggtacagacccgggttcgattcccggctggtgcaNNNNNNNNNNNNNNNNNNNNgtttcagagctatgctggaaacagcatagcaagttgaaataaggctagtccgttatcaacttgaaaaagtggcaccgagtcggtgcNNNNNNNNNNNNNNNNNNNNaaatattccgcggttctatctagttacgcgttaaaccaactagaa

>ZmUbi1 promoter
CTGCAGTGCAGCGTGACCCGGTCGTGCCCCTCTCTAGAGATAATGAGCATTGCATGTCTAAGTTATAAAAAATTACCACATATTTTTTTTGTCACACTTGTTTGAAGTGCAGTTTATCTATCTTTATACATATATTTAAACTTTACTCTACGAATAATATAATCTATAGTACTACAATAATATCAGTGTTTTAGAGAATCATATAAATGAACAGTTAGACATGGTCTAAAGGACAATTGAGTATTTTGACAACAGGACTCTACAGTTTTATCTTTTTAGTGTGCATGTGTTCTCCTTTTTTTTTGCAAATAGCTTCACCTATATAATACTTCATCCATTTTATTAGTACATCCATTTAGGGTTTAGGGTTAATGGTTTTTATAGACTAATTTTTTTAGTACATCTATTTTATTCTATTTTAGCCTCTAAATTAAGAAAACTAAAACTCTATTTTAGTTTTTTTATTTAATAATTTAGATATAAAATAGAATAAAATAAAGTGACTAAAAATTAAACAAATACCCTTTAAGAAATTAAAAAAACTAAGGAAACATTTTTCTTGTTTCGAGTAGATAATGCCAGCCTGTTAAACGCCGTCGACGAGTCTAACGGACACCAACCAGCGAACCAGCAGCGTCGCGTCGGGCCAAGCGAAGCAGACGGCACGGCATCTCTGTCGCTGCCTCTGGACCCCTCTCGAGAGTTCCGCTCCACCGTTGGACTTGCTCCGCTGTCGGCATCCAGAAATTGCGTGGCGGAGCGGCAGACGTGAGCCGGCACGGCAGGCGGCCTCCTCCTCCTCTCACGGCACCGGCAGCTACGGGGGATTCCTTTCCCACCGCTCCTTCGCTTTCCCTTCCTCGCCNGCCGTAATAAATAGACACCCCCTCCACACCCTCTTTCCCCAACCTCGTGTTGTTCGGAGCGCACACACACACAACCAGATCTCCCCCAAATCCACCCGTCGGCACCTCCGCTTCAAGGTACGCCGCTCGTCCTCCCCCCCCCTCTCTACCTTCTCTAGATCGGCGTTCCGGTCCATGGTTAGGGCCCGGTAGTTCTACTTCTGTTCATGTTTGTGTTAGATCCGTGTTTGTGTTAGATCCGTGCTGCTAGCGTTCGTACACGGATGCGACCTGTACGTCAGACACGTTCTGATTGCTAACTTGCCAGTGTTTCTCTTTGGGGAATCCTGGGATGGCTCTAGCCGTTCCGCAGACGGGATCGATTTCATGATTTTTTTTGTTTCGTTGCATAGGGTTTGGTTTGCCCTTTTCCTTTATTTCAATATATGCCGTGCACTTGTTTGTCGGGTCATCTTTTCATGCTTTTTTTTGTCTTGGTTGTGATGATGTGGTCTGGTTGGGCGGTCGTTCTAGATCGGAGTAGAATTCTGTTTCAAACTACCTGGTGGATTTATTAATTTTGGATCTGTATGTGTGTGCCATACATATTCATAGTTACGAATTGAAGATGATGGATGGAAATATCGATCTAGGATAGGTATACATGTTGATGCGGGTTTTACTGATGCATATACAGAGATGCTTTTTGTTCGCTTGGTTGTGATGATGTGGTGTGGTTGGGCGGTCGTTCATTCGTTCTAGATCGGAGTAGAATACTGTTTCAAACTACCTGGTGTATTTATTAATTTTGGAACTGTATGTGTGTGTCATACATCTTCATAGTTACGAGTTTAAGATGGATGGAAATATCGATCTAGGATAGGTATACATGTTGATGTGGGTTTTACTGATGCATATACATGATGGCATATGCAGCATCTATTCATATGCTCTAACCTTGAGTACCTATCTATTATAATAAACAAGTATGTTTTATAATTATTTTGATCTTGATATACTTGGATGATGGCATATGCAGCAGCTATATGTGGATTTTTTTAGCCCTGCCTTCATACGCTATTTATTTGCTTGGTACTGTTTCTTTTGTCGATGCTCACCCTGTTGTTTGGTGTTACTTCTGCAG

>AtUBQ10 promoter
AATACGAAATACGCTTCAATGCAGTGGGACCCACGGTTCAATTATTGCCAATTTTCAGCTCCACCGTATATTTAAAAAATAAAACGATAATGCTAAAAAAATATAAATCGTAACGATCGTTAAATCTCAACGGCTGGATCTTATGACGACCGTTAGAAATTGTGGTTGTCGACGAGTCAGTAATAAACGGCGTCAAAGTGGTTGCAGCCGGCACACACGAGTCGTGTTTATCAACTCAAAGCACAAATACTTTTCCTCAACCTAAAAATAAGGCAATTAGCCAAAAACAACTTTGCGTGTAAACAACGCTCAATACACGTGTCATTTTATTATTAGCTATTGCTTCACCGCCTTAGCTTTCTCGTGACCTAGTCGTCCTCGTCTTTTCTTCTTCTTCTTCTATAAAACAATACCCAAACAGCTCTTCTTCTTCACAATTCAGATTTCAATTTCTCAAAATCTTAAAAACTTTCTCTCAATTCTCTCTACCGTGATCAAGGTAAATTTCTGTGTTCCTTATTCTCTCAAAATCTTCGATTTTGTTTTCGTTCGATCCCAATTTCGTATATGTTCTTTGGTTTAGATTCTGTTAATCTTAGATCGAAGACGATTTTCTGGGTTTGATCGTTAGATATCATCTTAATTCTCGATTAGGGTTTCATAGATATCATCCGATTTGTTCAAATAATTTGAGTTTTGTCGAATAATTACTCTTCGATTTGTGATTTCTAACTAGATCTGGTGTTAGTTTCTAGTTTGTGCGATCGAATTTGTCGATTAATCTGAGTTTTTCTGATTAACAG

>ePPEmax

ATGAAGAGGACAGCCGATGGCAGCGAGTTCGAGAGCCCTAAGAAGAAGAGGAAGGTGGACAAGAAGTACTCGATCGGCCTCGATATTGGGACTAACTCTGTTGGCTGGGCCGTGATCACCGACGAGTACAAGGTGCCCTCAAAGAAGTTCAAGGTCCTGGGCAACACCGATCGGCATTCCATCAAGAAGAATCTCATTGGCGCTCTCCTGTTCGACAGCGGCGAGACGGCTGAGGCTACGCGGCTCAAGCGCACCGCCCGCAGGCGGTACACGCGCAGGAAGAATCGCATCTGCTACCTGCAGGAGATTTTCTCCAACGAGATGGCGAAGGTTGACGATTCTTTCTTCCACAGGCTGGAGGAGTCATTCCTCGTGGAGGAGGATAAGAAGCACGAGCGGCATCCAATCTTCGGCAACATTGTCGACGAGGTTGCCTACCACGAGAAGTACCCTACGATCTACCATCTGCGGAAGAAGCTCGTGGACTCCACAGATAAGGCGGACCTCCGCCTGATCTACCTCGCTCTGGCCCACATGATTAAGTTCAGGGGCCATTTCCTGATCGAGGGGGATCTCAACCCGGACAATAGCGATGTTGACAAGCTGTTCATCCAGCTCGTGCAGACGTACAACCAGCTCTTCGAGGAGAACCCCATTAATGCGTCAGGCGTCGACGCGAAGGCTATCCTGTCCGCTAGGCTCTCGAAGTCTCGGAAGCTCGAGAACCTGATCGCCCAGCTGCCGGGCGAGAAGAAGAACGGCCTGTTCGGGAATCTCATTGCGCTCAGCCTGGGGCTCACGCCCAACTTCAAGTCGAATTTCGATCTCGCTGAGGACGCCAAGCTGCAGCTCTCCAAGGACACATACGACGATGACCTGGATAACCTCCTGGCCCAGATCGGCGATCAGTACGCGGACCTGTTCCTCGCTGCCAAGAATCTGTCGGACGCCATCCTCCTGTCTGATATTCTCAGGGTGAACACCGAGATTACGAAGGCTCCGCTCTCAGCCTCCATGATCAAGCGCTACGACGAGCACCATCAGGATCTGACCCTCCTGAAGGCGCTGGTCAGGCAGCAGCTCCCCGAGAAGTACAAGGAGATCTTCTTCGATCAGTCGAAGAACGGCTACGCTGGGTACATTGACGGCGGGGCCTCTCAGGAGGAGTTCTACAAGTTCATCAAGCCGATTCTGGAGAAGATGGACGGCACGGAGGAGCTGCTGGTGAAGCTCAAGCGCGAGGACCTCCTGAGGAAGCAGCGGACATTCGATAACGGCAGCATCCCACACCAGATTCATCTCGGGGAGCTGCACGCTATCCTGAGGAGGCAGGAGGACTTCTACCCTTTCCTCAAGGATAACCGCGAGAAGATCGAGAAGATTCTGACTTTCAGGATCCCGTACTACGTCGGCCCACTCGCTAGGGGCAACTCCCGCTTCGCTTGGATGACCCGCAAGTCAGAGGAGACGATCACGCCGTGGAACTTCGAGGAGGTGGTCGACAAGGGCGCTAGCGCTCAGTCGTTCATCGAGAGGATGACGAATTTCGACAAGAACCTGCCAAATGAGAAGGTGCTCCCTAAGCACTCGCTCCTGTACGAGTACTTCACAGTCTACAACGAGCTGACTAAGGTGAAGTATGTGACCGAGGGCATGAGGAAGCCGGCTTTCCTGTCTGGGGAGCAGAAGAAGGCCATCGTGGACCTCCTGTTCAAGACCAACCGGAAGGTCACGGTTAAGCAGCTCAAGGAGGACTACTTCAAGAAGATTGAGTGCTTCGATTCGGTCGAGATCTCTGGCGTTGAGGACCGCTTCAACGCCTCCCTGGGGACCTACCACGATCTCCTGAAGATCATTAAGGATAAGGACTTCCTGGACAACGAGGAGAATGAGGATATCCTCGAGGACATTGTGCTGACACTCACTCTGTTCGAGGACCGGGAGATGATCGAGGAGCGCCTGAAGACTTACGCCCATCTCTTCGATGACAAGGTCATGAAGCAGCTCAAGAGGAGGAGGTACACCGGCTGGGGGAGGCTGAGCAGGAAGCTCATCAACGGCATTCGGGACAAGCAGTCCGGGAAGACGATCCTCGACTTCCTGAAGAGCGATGGCTTCGCGAACCGCAATTTCATGCAGCTGATTCACGATGACAGCCTCACATTCAAGGAGGATATCCAGAAGGCTCAGGTGAGCGGCCAGGGGGACTCGCTGCACGAGCATATCGCGAACCTCGCTGGCTCGCCAGCTATCAAGAAGGGGATTCTGCAGACCGTGAAGGTTGTGGACGAGCTGGTGAAGGTCATGGGCAGGCACAAGCCTGAGAACATCGTCATTGAGATGGCCCGGGAGAATCAGACCACGCAGAAGGGCCAGAAGAACTCACGCGAGAGGATGAAGAGGATCGAGGAGGGCATTAAGGAGCTGGGGTCCCAGATCCTCAAGGAGCACCCGGTGGAGAACACGCAGCTGCAGAATGAGAAGCTCTACCTGTACTACCTCCAGAATGGCCGCGATATGTATGTGGACCAGGAGCTGGATATTAACAGGCTCAGCGATTACGACGTCGATGCCATCGTTCCACAGTCATTCCTGAAGGATGACTCCATTGACAACAAGGTCCTCACCAGGTCGGACAAGAACCGGGGCAAGTCTGATAATGTTCCTTCAGAGGAGGTCGTTAAGAAGATGAAGAACTACTGGCGCCAGCTCCTGAATGCCAAGCTGATCACGCAGCGGAAGTTCGATAACCTCACAAAGGCTGAGAGGGGCGGGCTCTCTGAGCTGGACAAGGCGGGCTTCATCAAGAGGCAGCTGGTCGAGACACGGCAGATCACTAAGCACGTTGCGCAGATTCTCGACTCACGGATGAACACTAAGTACGATGAGAATGACAAGCTGATCCGCGAGGTGAAGGTCATCACCCTGAAGTCAAAGCTCGTCTCCGACTTCAGGAAGGATTTCCAGTTCTACAAGGTTCGGGAGATCAACAATTACCACCATGCCCATGACGCGTACCTGAACGCGGTGGTCGGCACAGCTCTGATCAAGAAGTACCCAAAGCTCGAGAGCGAGTTCGTGTACGGGGACTACAAGGTTTACGATGTGAGGAAGATGATCGCCAAGTCGGAGCAGGAGATTGGCAAGGCTACCGCCAAGTACTTCTTCTACTCTAACATTATGAATTTCTTCAAGACAGAGATCACTCTGGCCAATGGCGAGATCCGGAAGCGCCCCCTCATCGAGACGAACGGCGAGACGGGGGAGATCGTGTGGGACAAGGGCAGGGATTTCGCGACCGTCAGGAAGGTTCTCTCCATGCCACAAGTGAATATCGTCAAGAAGACAGAGGTCCAGACTGGCGGGTTCTCTAAGGAGTCAATTCTGCCTAAGCGGAACAGCGACAAGCTCATCGCCCGCAAGAAGGACTGGGATCCGAAGAAGTACGGCGGGTTCGACAGCCCCACTGTGGCCTACTCGGTCCTGGTTGTGGCGAAGGTTGAGAAGGGCAAGTCCAAGAAGCTCAAGAGCGTGAAGGAGCTGCTGGGGATCACGATTATGGAGCGCTCCAGCTTCGAGAAGAACCCGATCGATTTCCTGGAGGCGAAGGGCTACAAGGAGGTGAAGAAGGACCTGATCATTAAGCTCCCCAAGTACTCACTCTTCGAGCTGGAGAACGGCAGGAAGCGGATGCTGGCTTCCGCTGGCGAGCTGCAGAAGGGGAACGAGCTGGCTCTGCCGTCCAAGTATGTGAACTTCCTCTACCTGGCCTCCCACTACGAGAAGCTCAAGGGCAGCCCCGAGGACAACGAGCAGAAGCAGCTGTTCGTCGAGCAGCACAAGCATTACCTCGACGAGATCATTGAGCAGATTTCCGAGTTCTCCAAGCGCGTGATCCTGGCCGACGCGAATCTGGATAAGGTCCTCTCCGCGTACAACAAGCACCGCGACAAGCCAATCAGGGAGCAGGCTGAGAATATCATTCATCTCTTCACCCTGACGAACCTCGGCGCCCCTGCTGCTTTCAAGTACTTCGACACAACTATCGATCGCAAGAGGTACACAAGCACTAAGGAGGTCCTGGACGCGACCCTCATCCACCAGTCGATTACCGGCCTCTACGAGACGCGCATCGACCTGTCTCAGCTCGGGGGCGACTCCGGGAGCGAGACGCCAGGCACCTCCGAGTCGGCCACCCCAGAATCTGCCACAGTGGTGTCCGGCCAAAAGCAGGACCGCCAGGGCGGAGAACGCAGAAGGTCCCAGCTCGATAGGGATCAGTGTGCCTACTGCAAGGAGAAGGGCCACTGGGCCAAAGACTGCCCGAAAAAGCCGCGCGGCCCACGCGGCCCAAGGCCACAAACATCCCTCCTTCCAAAGAAGAAGCGGAAGGTGGAGCTCAGCGGAGGATCTTCCGGAGGATCTAGCGGCTCCGAGACACCAGGAACATCCGAAAGCGCTACACCAGAATCTAGCGGAGGCTCTTCCGGAGGATCTAGGCCTACCCTCAATATCGAGGACGAGTACAGGCTGCATGAGACATCCAAGGAGCCGGACGTGTCACTCGGCTCTACATGGCTGAGCGATTTCCCACAGGCCTGGGCGGAGACAGGCGGCATGGGCCTCGCGGTCAGGCAGGCGCCGCTCATCATTCCACTGAAGGCGACCTCCACACCGGTCAGCATCAAGCAGTACCCAATGTCACAGGAGGCACGGCTCGGCATCAAGCCACACATTCAGAGGCTCCTGGACCAGGGCATTCTGGTCCCTTGCCAGAGCCCGTGGAACACCCCTCTCCTGCCGGTGAAGAAGCCTGGCACAAATGACTACAGGCCGGTCCAGGATCTCAGGGAGGTGAACAAGCGCGTCGAGGATATCCATCCGACCGTGCCGAACCCATACAATCTCCTGTCAGGCCTCCCGCCATCTCACCAGTGGTACACCGTCCTCGACCTGAAGGATGCGTTCTTCTGCCTCAGGCTGCATCCAACAAGCCAGCCTCTCTTCGCCTTCGAGTGGCGCGATCCAGAGATGGGCATTTCAGGCCAGCTCACCTGGACACGGCTGCCACAGGGCTTCAAGAACTCTCCTACCCTCTTCAATGAGGCGCTCCATCGGGACCTGGCCGATTTCAGGATCCAGCACCCTGACCTCATTCTCCTGCAGTACGTGGACGATCTCCTGCTCGCCGCGACATCAGAGCTGGATTGCCAGCAGGGCACCAGGGCCCTGCTCCAGACACTCGGCAATCTGGGCTACCGGGCCTCTGCGAAGAAGGCCCAGATCTGCCAGAAGCAGGTGAAGTACCTCGGCTACCTGCTCAAGGAGGGACAGAGGTGGCTGACAGAGGCAAGGAAGGAGACAGTCATGGGCCAGCCTACCCCGAAGACACCTCGGCAGCTCAGGGAGTTCCTGGGCAAGGCCGGATTCTGCAGGCTCTTCATTCCAGGATTCGCGGAGATGGCGGCGCCACTCTACCCTCTGACCAAGCCGGGCACACTGTTCAACTGGGGCCCAGACCAGCAGAAGGCGTACCAGGAGATTAAGCAGGCACTGCTCACAGCACCTGCGCTCGGCCTGCCGGACCTCACAAAGCCATTCGAGCTGTTCGTGGATGAGAAGCAGGGCTACGCGAAGGGAGTCCTGACACAGAAGCTGGGACCATGGAGGCGCCCAGTGGCCTACCTCTCAAAGAAGCTCGACCCAGTGGCGGCCGGATGGCCTCCGTGCCTGAGGATGGTGGCGGCCATTGCCGTCCTCACCAAGGATGCCGGCAAGCTGACAATGGGCCAGCCTCTCGTGATTCTGGCGCCGCATGCGGTGGAGGCCCTGGTCAAGCAGCCACCTGATAGGTGGCTGTCCAACGCGCGCATGACCCACTACCAGGCCCTGCTCCTGGACACAGATAGGGTCCAGTTCGGACCAGTGGTGGCACTCAATCCTGCCACACTGCTGCCACTCCCTGAGGAGGGCCTGCAGCATAACTGCCTCGATATTCTGGCGGAGGCCCATGGCACCCGGCCAGACCTCACAGATCAGCCGCTGGACGATGACAAAATGGCACCGAAGAAAAAAAGGAAGGTCGGCGGCTCCCCGAAGAAAAAAAGGAAGGTCGGCGGCTCCCCGAAGAAAAAAAGGAAGGTCGGCGGCTCCCCGAAGAAAAAAAGGAAGGTCGGAATCCATGGCGGATCAGGATAG

>PEmax

ATGAAGAGGACAGCCGACGGCAGCGAGTTCGAGAGCCCTAAGAAGAAGCGTAAGGTGGACAAGAAGTACTCCATCGGCCTCGACATCGGCACTAACTCTGTGGGCTGGGCCGTGATCACCGACGAGTACAAGGTGCCCTCAAAGAAGTTCAAGGTCCTGGGCAACACCGACAGACACTCCATCAAGAAGAACCTCATCGGCGCTCTCCTGTTCGACAGCGGCGAGACTGCTGAGGCTACCAGACTCAAGAGAACCGCCAGACGTAGATACACCAGACGTAAGAACAGAATCTGCTACCTGCAGGAGATCTTCTCCAACGAGATGGCCAAGGTGGACGACTCATTCTTCCACAGGCTGGAGGAGTCATTCCTCGTCGAGGAGGACAAGAAGCACGAGAGACACCCAATCTTCGGCAACATCGTCGACGAGGTGGCCTACCACGAGAAGTACCCTACCATCTACCACCTGAGGAAGAAGCTCGTGGACTCCACAGACAAGGCCGACCTCAGACTGATCTACCTCGCTCTGGCCCACATGATCAAGTTCAGAGGCCACTTCCTGATCGAGGGCGACCTCAACCCTGACAACAGCGACGTGGACAAGCTGTTCATCCAGCTCGTGCAGACCTACAACCAGCTCTTCGAGGAGAACCCTATCAACGCCTCAGGCGTCGACGCTAAGGCTATCCTGTCCGCTAGACTCTCTAAGTCTAGGAAGCTCGAGAACCTGATCGCTCAGCTGCCTGGCGAGAAGAAGAACGGCCTGTTCGGCAACCTCATCGCTCTCAGCCTGGGCCTCACACCTAACTTCAAGTCCAACTTCGACCTCGCTGAGGACGCCAAGCTGCAGCTCTCCAAGGACACATACGACGACGACCTGGACAACCTCCTGGCCCAGATCGGCGACCAGTACGCCGACCTGTTCCTCGCTGCCAAGAACCTGTCCGACGCCATCCTCCTGTCTGACATCCTCAGGGTGAACACCGAGATCACCAAGGCTCCACTCTCAGCCTCCATGATCAAGAGATACGACGAGCACCACCAGGACCTGACCCTCCTGAAGGCCCTGGTCAGGCAGCAGCTCCCTGAGAAGTACAAGGAGATCTTCTTCGACCAGTCCAAGAACGGCTACGCTGGCTACATCGACGGCGGTGCCTCTCAGGAGGAGTTCTACAAGTTCATCAAGCCCATCCTGGAGAAGATGGACGGCACCGAGGAGCTGCTGGTGAAGCTCAAGCGTGAGGACCTCCTGAGGAAGCAGAGAACATTCGACAACGGCAGCATCCCACACCAGATCCACCTCGGCGAGCTGCACGCCATCCTGAGGAGGCAGGAGGACTTCTACCCATTCCTCAAGGACAACCGTGAGAAGATCGAGAAGATCCTGACCTTCAGGATCCCATACTACGTCGGCCCACTCGCTAGAGGCAACTCCAGATTCGCTTGGATGACCAGAAAGTCAGAGGAGACTATCACACCCTGGAACTTCGAGGAGGTGGTCGACAAGGGCGCTAGCGCTCAGTCCTTCATCGAGAGGATGACCAACTTCGACAAGAACCTGCCAAACGAGAAGGTGCTCCCTAAGCACTCCCTGCTGTACGAGTACTTCACCGTCTACAACGAGCTGACCAAGGTGAAGTATGTGACCGAGGGCATGAGGAAGCCCGCTTTCCTGTCTGGCGAGCAGAAGAAGGCCATCGTGGACCTCCTGTTCAAGACCAACAGAAAGGTCACCGTGAAGCAGCTCAAGGAGGACTACTTCAAGAAGATCGAGTGCTTCGACTCCGTCGAGATCTCTGGCGTGGAGGACAGATTCAACGCCTCCCTGGGCACCTACCACGACCTCCTGAAGATCATCAAGGACAAGGACTTCCTGGACAACGAGGAGAACGAGGACATCCTCGAGGACATCGTGCTGACACTCACTCTGTTCGAGGACAGAGAGATGATCGAGGAGCGTCTGAAGACTTACGCCCACCTCTTCGACGACAAGGTCATGAAGCAGCTCAAGAGGCGTAGGTACACCGGCTGGGGCAGGCTGAGCAGGAAGCTCATCAACGGCATCAGAGACAAGCAGTCCGGCAAGACCATCCTCGACTTCCTGAAGAGCGACGGCTTCGCCAACAGAAACTTCATGCAGCTGATCCACGACGACAGCCTCACATTCAAGGAGGACATCCAGAAGGCTCAGGTGAGCGGCCAGGGCGACTCCCTGCACGAGCACATCGCCAACCTCGCTGGCTCACCAGCTATCAAGAAGGGCATCCTGCAGACCGTGAAGGTGGTGGACGAGCTGGTGAAGGTCATGGGCAGGCACAAGCCTGAGAACATCGTCATCGAGATGGCCAGAGAGAACCAGACCACCCAGAAGGGCCAGAAGAACTCACGTGAGAGGATGAAGAGGATCGAGGAGGGCATCAAGGAGCTGGGCTCCCAGATCCTCAAGGAGCACCCTGTGGAGAACACCCAGCTGCAGAACGAGAAGCTCTACCTGTACTACCTCCAGAACGGCAGAGACATGTATGTGGACCAGGAGCTGGACATCAACAGGCTCAGCGACTACGACGTCGACGCCATCGTGCCACAGTCATTCCTGAAGGACGACTCCATCGACAACAAGGTCCTCACCAGGTCCGACAAGAACAGAGGCAAGTCTGACAACGTGCCTTCAGAGGAGGTCGTGAAGAAGATGAAGAACTACTGGAGACAGCTCCTGAACGCCAAGCTGATCACCCAGAGAAAGTTCGACAACCTCACAAAGGCTGAGAGAGGCGGCCTCTCTGAGCTGGACAAGGCCGGCTTCATCAAGAGGCAGCTGGTCGAGACAAGACAGATCACTAAGCACGTGGCCCAGATCCTCGACTCAAGGATGAACACTAAGTACGACGAGAACGACAAGCTGATCAGAGAGGTGAAGGTCATCACCCTGAAGTCAAAGCTCGTGTCCGACTTCAGGAAGGACTTCCAGTTCTACAAGGTGAGAGAGATCAACAACTACCACCACGCCCACGACGCCTACCTGAACGCCGTGGTCGGCACAGCTCTGATCAAGAAGTACCCAAAGCTCGAGAGCGAGTTCGTGTACGGCGACTACAAGGTGTACGACGTGAGGAAGATGATCGCCAAGTCCGAGCAGGAGATCGGCAAGGCTACCGCCAAGTACTTCTTCTACTCTAACATCATGAACTTCTTCAAGACAGAGATCACTCTGGCCAACGGCGAGATCCGTAAGAGGCCACTCATCGAGACTAACGGCGAGACTGGCGAGATCGTGTGGGACAAGGGCAGGGACTTCGCCACCGTCAGGAAGGTGCTCTCCATGCCACAGGTGAACATCGTCAAGAAGACAGAGGTCCAGACTGGCGGATTCTCTAAGGAGTCAATCCTGCCTAAGAGAAACAGCGACAAGCTCATCGCCCGTAAGAAGGACTGGGACCCTAAGAAGTACGGCGGATTCGACAGCCCTACTGTGGCCTACTCCGTCCTGGTGGTGGCCAAGGTCGAGAAGGGCAAGTCCAAGAAGCTCAAGAGCGTGAAGGAGCTGCTGGGCATCACCATCATGGAGAGATCCAGCTTCGAGAAGAACCCTATCGACTTCCTGGAGGCCAAGGGCTACAAGGAGGTCAAGAAGGACCTGATCATCAAGCTCCCTAAGTACTCACTCTTCGAGCTGGAGAACGGCAGGAAGCGTATGCTGGCTTCCGCTGGCGAGCTGCAGAAGGGCAACGAGCTGGCTCTGCCCTCCAAGTATGTGAACTTCCTCTACCTGGCCTCCCACTACGAGAAGCTCAAGGGCAGCCCTGAGGACAACGAGCAGAAGCAGCTGTTCGTCGAGCAGCACAAGCACTACCTCGACGAGATCATCGAGCAGATCTCCGAGTTCTCCAAGAGAGTGATCCTGGCCGACGCCAACCTGGACAAGGTCCTCTCCGCCTACAACAAGCACAGAGACAAGCCAATCAGGGAGCAGGCTGAGAACATCATCCACCTCTTCACCCTGACCAACCTCGGCGCTCCTGCTGCTTTCAAGTACTTCGACACAACTATCGACCGTAAGAGGTACACAAGCACCAAGGAGGTCCTGGACGCCACCCTCATCCACCAGTCCATCACCGGCCTCTACGAGACTAGAATCGACCTGTCTCAGCTCGGCGGCGACTCAGGCGGATCATCTGGCGGATCAAAGAGAACAGCCGACGGCTCTGAGTTCGAGTCACCTAAGAAGAAGCGTAAGGTGTCAGGCGGCTCTTCAGGCGGCAGCACCCTGAACATTGAGGACGAGTACAGACTGCACGAGACAAGCAAGGAGCCAGACGTGTCACTCGGTAGCACTTGGCTCTCTGACTTCCCACAGGCTTGGGCCGAGACTGGTGGTATGGGTCTGGCCGTGAGACAGGCTCCACTGATCATCCCTCTGAAGGCTACCTCCACACCAGTGTCTATTAAGCAGTACCCAATGAGCCAGGAGGCCAGGCTGGGAATCAAGCCACACATTCAGAGACTGCTGGACCAGGGTATCCTGGTGCCATGCCAGTCACCCTGGAATACTCCACTCCTGCCAGTGAAGAAGCCTGGAACAAACGACTACAGGCCAGTGCAGGATCTCAGGGAGGTGAACAAGAGAGTGGAGGACATCCATCCAACAGTGCCAAACCCATACAATCTGCTGTCAGGTCTGCCTCCAAGCCACCAGTGGTACACCGTCCTGGACCTCAAGGACGCCTTCTTCTGCCTGAGACTGCACCCAACATCTCAGCCACTGTTCGCTTTCGAGTGGAGAGATCCAGAGATGGGTATTTCCGGTCAGCTGACCTGGACAAGACTGCCACAGGGTTTCAAGAACTCACCAACTCTCTTCAACGAGGCTCTCCACAGAGATCTCGCTGACTTCAGGATTCAGCATCCCGATCTGATCCTGCTCCAGTATGTGGACGACCTGCTGCTCGCCGCTACATCAGAGCTGGACTGCCAGCAGGGTACCAGAGCCCTGCTGCAGACACTGGGTAATCTGGGATACAGAGCCTCTGCTAAGAAGGCTCAGATCTGCCAGAAGCAGGTCAAGTACCTGGGTTACCTGCTCAAGGAGGGTCAGAGATGGCTCACTGAGGCTCGTAAGGAGACTGTGATGGGTCAGCCCACTCCAAAGACTCCAAGGCAGCTCAGGGAGTTCCTCGGCAAGGCTGGATTCTGCAGACTGTTCATCCCTGGATTCGCTGAGATGGCTGCTCCACTCTACCCACTGACTAAGCCAGGAACACTGTTCAACTGGGGACCAGACCAGCAGAAGGCTTACCAGGAGATTAAGCAGGCTCTGCTGACAGCTCCAGCTCTCGGTCTGCCAGACCTGACAAAGCCATTCGAGCTGTTCGTGGACGAGAAGCAGGGATACGCTAAGGGTGTGCTGACACAGAAGCTGGGACCTTGGAGAAGACCAGTCGCTTACCTGTCAAAGAAGCTGGACCCAGTCGCTGCTGGATGGCCTCCATGCCTCAGAATGGTCGCTGCTATTGCTGTGCTGACCAAGGATGCTGGAAAGCTCACAATGGGACAGCCTCTCGTGATCCTGGCTCCACATGCTGTGGAGGCTCTGGTGAAGCAGCCACCAGACAGATGGCTGTCAAACGCTAGAATGACACACTACCAGGCTCTCCTGCTCGATACAGACAGAGTGCAGTTCGGACCTGTGGTGGCTCTGAACCCAGCCACACTGCTGCCACTCCCTGAGGAGGGACTCCAGCACAATTGCCTCGACATCCTGGCTGAGGCTCACGGTACCAGACCTGATCTCACCGACCAGCCTCTGCCAGATGCTGACCACACCTGGTACACAGATGGATCCTCACTGCTGCAGGAGGGTCAGCGTAAGGCTGGTGCCGCCGTCACCACAGAGACAGAGGTCATTTGGGCCAAGGCCCTGCCAGCTGGTACCAGCGCCCAGAGAGCTGAGCTGATCGCTCTGACTCAGGCTCTGAAGATGGCCGAGGGCAAGAAGCTCAATGTGTACACCGACTCAAGATACGCTTTCGCTACAGCTCACATTCATGGAGAGATCTACAGACGTAGAGGATGGCTGACTTCCGAGGGCAAGGAGATTAAGAATAAGGACGAGATCCTGGCCCTGCTCAAGGCCCTGTTCCTGCCAAAGAGACTCTCAATCATTCACTGCCCTGGTCACCAGAAGGGTCACTCAGCCGAGGCTAGAGGTAATAGAATGGCTGACCAGGCTGCTAGAAAGGCTGCTATCACCGAGACTCCCGATACATCTACCCTCCTGATCGAGAACTCAAGCCCATCTGGCGGATCTAAGAGAACTGCCGACGGATCTGAGTTCGAGTCACCTAAGAAGAAGCGTAAGGTGGGATCTGGACCTGCCGCCAAGAGAGTGAAGCTCGACTGA

Table S2. Prime editing targets sequences used in this study

|  | **PE targets** | **Nicking targets** | **Distance to PE target** | **RT+PBS** |
| --- | --- | --- | --- | --- |
| *EPSPS* | GCAGTAACTGCAGCTGCCAATGG | CCCAGTGGCGAAAGAATCAAGGG | 59 bp | TGGAATAGTCATGCGTTCATTGGCAGCTGCA |
| *ALS* | ATCTAGGCATGGTTGTGCAGTGG | AATATCTCGGTCTCGTTTGATGG | 60 bp | CCGATCTTCCAGCTGCACAACCATG |
| *AGA2* | CTGACCCCAGGATCAGGCCTTGG | ACTTTTACTGTTCGAAGCAGTGG | 38 bp | AACAAGTCACAGTCCTGATCCT |
| *WIP1* | ACTACTACTGGACTTAATAAGGG | AGAGGGCAAGAAAACTGAGTTGG | 52 bp | ATATTGACATTTGTTAAGTCCAGT |

Table S3. Next-generation sequencing (NGS) analysis of desired prime editing (PE) efficiency in T0 transgenic plants.

|  | Desired PE efficiency | |
| --- | --- | --- |
| Individual T0 Plant | pPE002-EPSPS | pPE003-EPSPS |
| 1 | 52.02% | 55.34% |
| 2 | 38.88% | 48.09% |
| 3 | 25.37% | 27.15% |
| 4 | 21.91% | 23.74% |
| 5 | 12.40% | 16.67% |
| 6 | 10.79% | 13.26% |
| 7 | 10.23% | 11.22% |
| 8 | 8.67% | 10.93% |
| 9 | 6.42% | 9.12% |
| 10 | 5.80% | 8.65% |
| 11 | 0.00% | 7.81% |
| 12 | 0.00% | 6.74% |
| 13 | 0.00% | 0.00% |
| 14 | 0.00% | 0.00% |
| ... | 0.00% | 0.00% |
| Total analysed plants | 47 | 57 |
| Average PE efficiency | 4.10% | 4.19% |

The "Desired PE efficiency" represents the percentage of NGS reads containing the precise intended *TAP-IVS* edits at the *EPSPS* target site among the total aligned reads for each individual plant. A total of 47 and 57 independent T0 plants were analyzed for the pPE002-EPSPS and pPE003-EPSPS vectors, respectively. The ellipsis (...) indicates that the remaining analyzed plants for each vector showed 0% desired editing efficiency. The "Average PE efficiency" is calculated as the mean efficiency across all analyzed plants for each construct.

Table S4 Genotype segregation of the T2 progeny derived from non-transgenic heterozygous T1 plants.

| Genotype | Number of Plants (n) | Percentage (%) | Expected Mendelian Ratio |
| --- | --- | --- | --- |
| Wild-type (WT) | 181 | 36.2% | 25% |
| Heterozygous (He) | 295 | 59.0% | 50% |
| Homozygous (Ho) | 24 | 4.8% | 25% |
| Total | 500 | 100% | 100% |

The observed segregation ratio deviated highly significantly from the expected 1:2:1 Mendelian ratio (X^2^ = 114.80, *P* < 0.0001, Chi-square test), indicating a severe fitness cost or partial lethality associated with the homozygous EPSPS mutation.

Table S5 Effects of prime editing and glyphosate treatment on key agronomic traits.

| Genotype and Treatment | Fruit Weight (g) | Soluble Solids Content (%) | Seed Number per Fruit |
| --- | --- | --- | --- |
| Wild-type (Untreated) | 1980.0 ± 246.5 a | 10.4 ± 0.7 a | 202.8 ± 17.5 a |
| Heterozygous (Untreated) | 2020.0 ± 270.7 a | 10.0 ± 0.9 a | 197.8 ± 21.8 a |
| Heterozygous (1× Glyphosate) | 1880.0 ± 152.5 a | 10.0 ± 1.1 a | 199.6 ± 22.6 a |

Data are presented as the mean ± standard deviation (SD) from five biological replicates (n = 5). Identical letters (a) indicate no significant differences among the different genotypes and treatments (*P* > 0.05, one-way ANOVA followed by Tukey's HSD test).

Table S6. Primers for amplification of prime editing targets in this study.

| **Primer name** | **Sequence (5'-3')** |
| --- | --- |
| EPSPS-JD-F | CCGCGATTTATCATCCAGTA |
| EPSPS-JD-R | ATCTGCACCAAGCTGCTTGA |
| ALS-JD-F | GATGTGGGCAGCTCAGTTCTAC |
| ALS-JD-R | AGTATACGGATCTTCCATCTCC |
| AGA2-JD-F | GCGCAGCAACTGTTTGTTGCTC |
| AGA2-JD-R | CGGAAACAACACAGGAAGCGGT |
| WIP1-JD-F | CCTGCAAGAGTAGCACAAGAT |
| WIP1-JD-R | CCTAGTCACTCCCAAGTTATCA |
